# Statistical methods for the analysis of adverse event data in randomised controlled trials: a scoping review and taxonomy

**DOI:** 10.1186/s12874-020-01167-9

**Published:** 2020-11-30

**Authors:** Rachel Phillips, Odile Sauzet, Victoria Cornelius

**Affiliations:** 1 Imperial Clinical Trials Unit, Imperial College London, 1st Floor Stadium House, 68 Wood Lane, London, W12 7RH United Kingdom; 2 School of Public Health / AG 3 Epidemiologie & International Public Health, Bielefeld University, Bielefeld, Germany

**Keywords:** Randomised controlled trials, Adverse events, harms, adverse drug reactions, Scoping review, Methodological review, Investigational drug, Signal detection

## Abstract

**Background:**

Statistical methods for the analysis of harm outcomes in randomised controlled trials (RCTs) are rarely used, and there is a reliance on simple approaches to display information such as in frequency tables. We aimed to identify whether any statistical methods had been specifically developed to analyse prespecified secondary harm outcomes and non-specific emerging adverse events (AEs).

**Methods:**

A scoping review was undertaken to identify articles that proposed original methods or the original application of existing methods for the analysis of AEs that aimed to detect potential adverse drug reactions (ADRs) in phase II-IV parallel controlled group trials. Methods where harm outcomes were the (co)-primary outcome were excluded.

Information was extracted on methodological characteristics such as: whether the method required the event to be prespecified or could be used to screen emerging events; and whether it was applied to individual events or the overall AE profile. Each statistical method was appraised and a taxonomy was developed for classification.

**Results:**

Forty-four eligible articles proposing 73 individual methods were included. A taxonomy was developed and articles were categorised as: visual summary methods (8 articles proposing 20 methods); hypothesis testing methods (11 articles proposing 16 methods); estimation methods (15 articles proposing 24 methods); or methods that provide decision-making probabilities (10 articles proposing 13 methods). Methods were further classified according to whether they required a prespecified event (9 articles proposing 12 methods), or could be applied to emerging events (35 articles proposing 61 methods); and if they were (group) sequential methods (10 articles proposing 12 methods) or methods to perform final/one analyses (34 articles proposing 61 methods).

**Conclusions:**

This review highlighted that a broad range of methods exist for AE analysis. Immediate implementation of some of these could lead to improved inference for AE data in RCTs. For example, a well-designed graphic can be an effective means to communicate complex AE data and methods appropriate for counts, time-to-event data and that avoid dichotomising continuous outcomes can improve efficiencies in analysis. Previous research has shown that adoption of such methods in the scientific press is limited and that strategies to support change are needed.

**Trial registration:**

PROSPERO registration: https://www.crd.york.ac.uk/prospero/display_record.php?RecordID=97442

**Supplementary Information:**

The online version contains supplementary material available at 10.1186/s12874-020-01167-9.

## Background

Randomised controlled trials (RCTs) are considered the ‘gold-standard’ for evaluating the efficacy/effectiveness of interventions. RCTs also provide invaluable information to allow evaluation of the harm profile of interventions. The comparator arm provides an opportunity to compare rates of adverse events (AEs) which enables signals for potential adverse drug reactions (ADRs) to be identified [1, 2].^1^ Whilst statistical analysis methods for efficacy outcomes in clinical trials are well established the same cannot be said for the analysis of harm outcomes [3–5].

The last 15 years has seen increasing emphasis on developing harm profiles of drugs. Working groups have developed guidance on the reporting of harm data for journal articles. Including: the harms extension to CONSORT; the pharmaceutical industry standard from the Safety Planning, Evaluation and Reporting Team (SPERT); the extension of PRISMA for harms reporting in systematic reviews; and the joint pharmaceutical/journal editor collaboration guidance on reporting of harm data in journal articles [6–9]. Regulators including the European Commission and the Food and Drug Administration have also issued detailed guidance on the collection and presentation of AEs/Rs arising in clinical trials [10–12]. Whilst these recommendations and guidelines call for better practice in collection and reporting, they are limited in recommendations for improving statistical analysis practices. The pharmaceutical industry standard from SPERT has perhaps given the greatest consideration to analytical approaches, for example suggesting consideration should be given to survival techniques [9].

Analysing harms in RCTs is not without its challenges and could, in part, explain a lack of progress in analysis practices [13, 14]. Unlike efficacy outcomes which are well defined and restricted in number at the planning stage of an RCT, we collect numerous, undefined harms in RCTs. Furthermore, collection requires additional information to be obtained on factors such as severity, timing and duration, number of occurrences and outcome, which for efficacy outcomes would have all been predefined [4]. From a statistical perspective consideration to type-I (false-positive) and type-II (false-negative) errors is crucial especially when considering how to analyse non-pre-specified emerging events. RCTs are typically designed to test the efficacy of an intervention and are not powered to detect differences in harm outcomes such as detecting difference in proportions of events, which could be indicative of an ADR. As a trial is not powered to detect ADRs, there is a possibility that any statistical testing of data may result in the drug being deemed safe or a trial not being stopped early enough resulting in more participants than necessary suffering an ADR. In addition, the vast number of potential emerging events can lead to issues of multiplicity [15, 16]. That said any adjustment for multiplicity is likely to make a “finding untenable” and therefore the value of adopting traditional sequential monitoring methods used for efficacy outcomes might be limited for monitoring harms [17]. It is also important to consider the impact of differential follow-up and/or exposure times, the time events occur and dependencies between events and analysis should account for this where necessary [18].

Despite these complexities journal articles, one of the main sources of dissemination of clinical trial results, predominantly rely on simple approaches such as tables of frequencies and percentages when reporting AEs [4, 19]. In view of the lack of sophisticated statistical methods used for the analysis of harm outcomes we performed a review to investigate which statistical methods have been proposed in order to improve awareness and facilitate their use.

## Methods

### Aim

To identify and classify statistical methods that have been specifically developed or adapted for use in RCTs to analyse prespecified secondary harm outcomes and non-specific emerging AEs. We undertook a scoping review to identify methods for AE analysis in RCTs whose aim was to flag signals for potential ADRs. A scoping review was conducted to uncover all proposed methodology rather than a more structured systematic review as we did not aim to perform a quantitative synthesis and did not want to limit the scope of our results [20].

### Search strategy

A systematic search of Medline and Embase databases via Ovid and the Web of Science and Scopus databases was performed in March 2018 and updated up until October 2019. No time restrictions were placed on the search. The search strategy was developed by studying key references in consultation with both experts in the field and experts in review methodology. Full details of the search terms can be found in Additional file 1. Reference lists of all eligible articles were also searched and a search of the Web of Science database was undertaken to identify citations of included articles.

One reviewer (RP) screened titles and abstracts of articles identified. Full text articles were scrutinised for eligibility and all queries regarding eligibility were discussed with at least one other reviewer (VC or OS).

### Selection criteria

The review included articles that proposed original methods or the original application of existing methods developed for the analysis of AEs in phase II-IV trials that aimed to identify potential ADRs in a parallel controlled group setting. Methods where harm outcomes were the primary or co-primary outcome such as dose-finding or risk-benefit methods were excluded. Established methods designed to monitor efficacy outcomes, which could be used to monitor prespecified harm outcomes, such as the methods of e.g. O’Brien and Fleming, Lan and DeMets, were excluded [21, 22]. Foreign language articles were translated where needed. Full eligibility criteria is specified in the review protocol, which can be accessed via the PROSPERO register for systematic reviews (https://www.crd.york.ac.uk/prospero/display_record.php?RecordID=97442).

### Data extraction

Data from eligible articles was extracted using a standardised pre-piloted data extraction form (RP) (Additional file 2). Information was collected on methodological characteristics including: whether the method required the event to be prespecified or could be used to screen emerging events; whether it was applied to individual events or the overall adverse event profile; data type applicable to e.g. continuous, proportion, count, time-to-event; whether any test was performed; what, if any, assumptions were made; if any prior or external information could be incorporated; and what the output included e.g. summary statistic, test-statistic, *p*-value, plot etc. All queries were discussed with a second reviewer (VC) and clarified with a third reviewer (OS), if necessary.

### Analysis

Results are reported as per the PRISMA extension for scoping reviews [23, 24]. Each statistical method was appraised in turn and a taxonomy was developed for classification. Data analysis was primarily descriptive, and methods are summarised and presented by taxonomy.

## Results

### Study selection

**Fig. 1:**
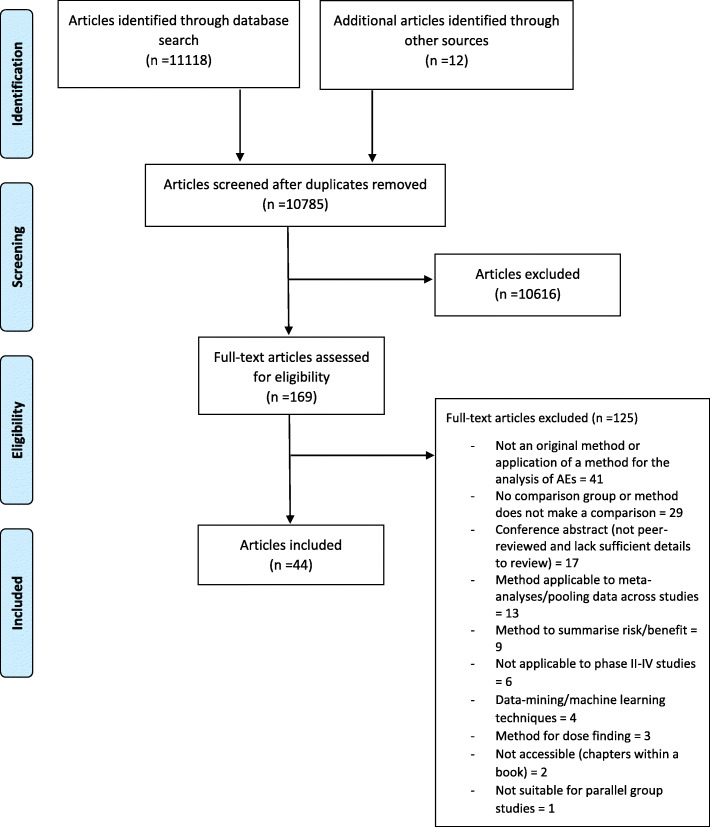
Flow diagram describing the assessment of sources of evidence

The search identified 11,118 articles. After duplicate articles were removed, 10,773 articles were screened, 10 articles were identified from the reference lists of eligible articles and two articles were identified through the search of citations of eligible articles. Review of titles and abstracts reduced the number of articles for full review to 169. Review of full text articles resulted in a further 125 exclusions (Additional file 3 lists the articles excluded at this point). The main reasons for exclusion after full text review were: the method presented was not original or the original application of a method for the analysis of AEs (33%); there was no comparison group or comparison made (23%); articles were published conference abstracts and therefore were not peer-reviewed and/or lacked sufficient detail to undergo a full review (14%). This left 44 eligible articles for inclusion that proposed 73 individual methods (Fig. 1).

### Characteristics of articles

Articles were predominantly published by authors working in industry (*n* = 20 (45%)), eight (18%) were published by academic authors and four (9%) were published by authors from the public sector. Eight (18%) articles were from an industry/academic collaboration, two (5%) an academic/public sector collaboration, one (2%) an industry/public sector collaboration and one (2%) from an industry/academic/public sector collaboration.

### Taxonomy of statistical methods for AE analysis

**Fig. 2:**
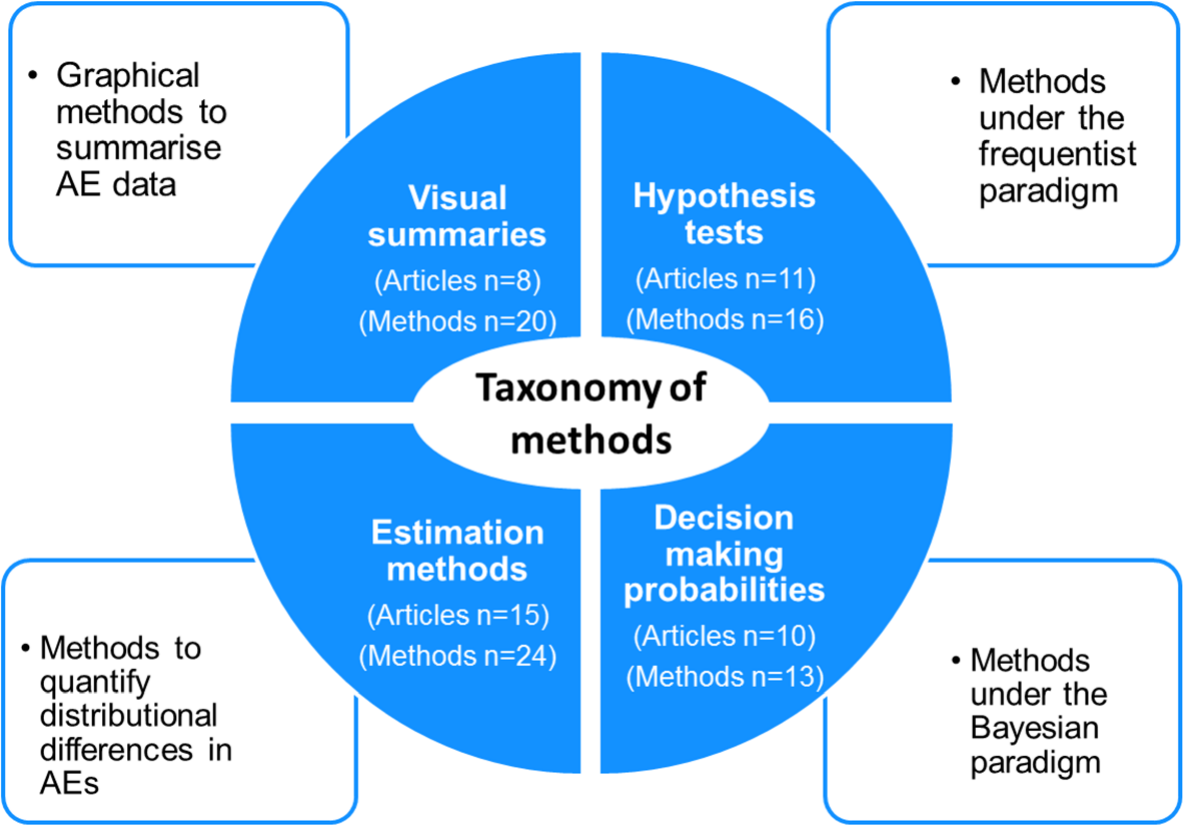
Taxonomy of methods for adverse event (AE) analysis

Due to the number and variety of methods identified, we developed a taxonomy to classify methods. Four groups were identified (Fig. 2).

#### Visual summary methods

Methods that propose graphical approaches to view single or multiple AEs as the principal analysis method.

#### Hypothesis testing methods

Methods under the frequentist paradigm. These methods set up a testable hypothesis and use evidence against the null hypothesis in terms of *p*-values based on the data observed in the current trial.

#### Estimation methods

Methods that quantify distributional differences in AEs between treatment groups without a formal test.

#### Methods that provide decision making probabilities

Statistical methods under the Bayesian paradigm. The overarching characteristic of these methods is output of (posterior) predicted probabilities regarding the chance of a predefined threshold of risk being exceeded based on the data observed in the current trial and/or any relevant prior knowledge.

**Fig. 3:**
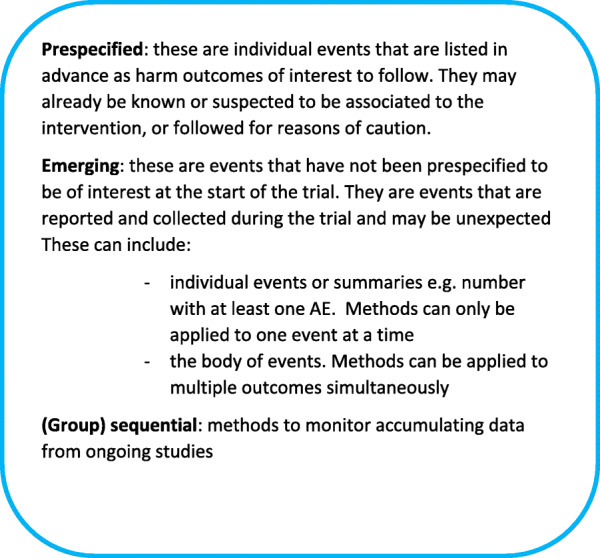
Classification terminology

All methods were further sub-divided into whether they were for use on prespecified events, which could be listed in advance as harm outcomes of interest to follow-up and may already be known or suspected to be associated with the intervention, or followed for reasons of caution; or could be applied to emerging (not prespecified) events that are reported and collected during the trial and may be unexpected. Further, we made the distinction between (group) sequential methods (methods to monitor accumulating data from ongoing studies) and methods for final/one analysis (Fig. 3).

The number of articles and methods identified by type is provided in Table 1. Articles most frequently proposed estimation methods (15 articles proposing 24 methods), followed by hypothesis testing methods (11 articles proposing 16 methods). Ten articles proposed 13 methods to provide decision-making probabilities and eight articles proposed 20 visual summaries. The majority of articles developed methods for emerging events (35 articles proposing 61 methods) and final/one analysis (34 articles proposing 61 methods). Individual article classifications and brief summaries are presented in Table 2 and articles ranked according to ease of comprehension/implementation are provided in Additional file 4.

**Table 1:** Summary level classifications

	Taxonomy of methods			
	Visual Articles *N* = 8 [Methods N = 20]	Hypothesis testing Articles *N* = 11 [Methods *N* = 16]	Estimation Articles *N* = 15 [Methods *N* = 24]	Decision making probabilities Articles *N* = 10 [Methods *N* = 13]
Classification	n (%)	n (%)	n (%)	n (%)
Type of event				
Prespecified	0 (0) [0 (0)]	5 (55.6) [7 (58.3)]	0 (0) [0 (0)]	4 (44.4) [5 (41.7)]
Emerging	8 (22.9) [20 (32.8)]	6 (17.1) [9 (14.8)]	15 (42.9) [24 (39.3)]	6 (17.1) [8 (13.1)]
Time of analysis				
(Group) sequential	0 (0) [0 (0)]	5 (50.0) [6 (50.0)]	0 (0) [0 (0)]	5 (50.0) [6 (50.0)]
Final/one-analysis	8 (23.5) [20 (32.8)]	6 (17.6) [10 (16.4)]	15 (44.1) [24 (37.5)]	5 (14.7) [7 (11.5)]

**Table 2:** Article classifications

Authors	Year	Taxonomy ^a^	Further classification variables		Brief summary
		V, HT, E, DMP	Prespecified or Emerging (single or multiple outcomes)	(Group) Sequential (monitoring) - yes/no	
Amit, Heiberger & Lane [25]	2008	V	Emerging (single & multiple)	No	Dot plot for emerging AEs, Kaplan-Meier and hazard function for single AEs and cumulative frequency plots, boxplots and line graphs for continuous outcomes
Chuang-Stein, Le & Chen [26]	2001	V	Emerging (single)	No	Displays two-by-two frequencies graphically for emerging AEs, histograms and delta plots for continuous outcomes
Chuang-Stein & Xia [27]	2013	V	Emerging (single & multiple)	No	Bar charts, Venn diagrams and Forest plots for emerging AEs, risk over time for single AEs and e-Dish plots for continuous outcomes
Karpefors & Weatherall [28]	2018	V	Emerging (multiple)	No	Tendril plot for emerging AEs
Southworth [29]	2008	V	Emerging (single)	No	Scatterplot with regression outputs for continuous outcomes
Trost & Freston [30]	2008	V	Emerging (multiple)	No	Vector plots for continuous outcomes, includes 3 outcomes per plot
Zink, Wolfinger & Mann [31]	2013	V	Emerging (multiple)	No	Volcano plot for emerging AEs
Zink, Marchenko, Sanchez-Kam, Ma & Jiang [14]	2018	V	Emerging (multiple)	No	Heat map for emerging AEs
Bolland & Whitehead [32]	2000	HT	Prespecified	Yes	Alpha spending function
Fleishman & Parker [33]	2012	HT	Prespecified	Yes	Alpha spending function, adjustment to significance threshold and conditional power
Lieu et al. [34]	2007	HT	Prespecified	Yes	Likelihood ratio test
Liu [35]	2007	HT	Prespecified	No	Non-inferiority test
Shih, Lai, Heyse & Chen [36]	2010	HT	Prespecified	Yes	Likelihood ratio test
Agresti & Klingenberg [37]	2005	HT	Emerging (overall profile)	No	Multivariate likelihood ratio tests for overall AE numbers
Bristol & Patel [38]	1990	HT	Emerging (overall profile)	No	Multivariate likelihood ratio test with Markov chains for overall AE numbers, incorporating recurrent events
Chuang-Stein, Mohberg & Musselman [39]	1992	HT	Emerging (overall profile)	No	Multivariate test for overall AE numbers with chi-squared distribution, incorporating severity and participant acceptability scores
Huang, Zalkikar & Tiwari [40]	2014	HT	Emerging (single)	Yes	Likelihood ratio tests for AE rate (i.e. incorporating exposure time), incorporating recurrent events
Mehrotra & Adewale [41]	2012	HT	Emerging (multiple)	No	P-value adjustment
Mehrotra & Heyse [42]	2004	HT	Emerging (multiple)	No	P-value adjustment
Allignol, Beyersmann & Schmoor [43]	2016	E	Emerging (single)	No	Estimates cumulative incidence function in presence of competing risks
Borkowf [44]	2006	E	Emerging (single)	No	Confidence interval for difference in proportions
Evans & Nitsch [45]	2012	E	Emerging (single)	No	Proportions, incidences, odds ratios etc.
Gong, Tong, Strasak & Fang [46]	2014	E	Emerging (single)	No	Non-parametric estimate for mean cumulative number of recurrent events in presence of competing risks
Hengelbrock, Gillhaus, Kloss & Leverkus [47]	2016	E	Emerging (single)	No	Survival based methods to estimate hazard ratios for recurrent events
Lancar, Kramar & Haie-Meder [48]	1995	E	Emerging (single)	No	Non-parametric estimate for prevalence allowing for recurrent events
Leon-Novelo, Zhou, Nebiyou Bekele & Muller [49]	2010	E	Emerging (multiple)	No	Bayesian approach to estimate the probability of severity grading of events in treatment and control groups separately
Liu, Wang, Liu & Snavely [50]	2006	E	Emerging (single)	No	Confidence interval for difference in exposure adjusted incidence rates
Nishikawa, Tango & Ogawa [51]	2006	E	Emerging (single)	No	Estimates the cumulative incidence function in presence of competing risks and conditional estimate for recurrent events
O’Gorman, Woolson & Jones [52]	1994	E	Emerging (single)	No	Confidence intervals for difference in proportion
Rosenkranz [53]	2006	E	Emerging (single)	No	Survival based method to estimate dependence between AE time and discontinuation time
Siddiqui [15]	2009	E	Emerging (single)	No	Non-parametric estimate for the cumulative mean number of events allowing for recurrent events
Sogliero-Gilbert, Ting, & Zubkoff [54]	1991	E	Emerging (multiple)	No	A score to indicate abnormal laboratory values
Wang & Quartey [55]	2012	E	Emerging (single)	No	Non-parametric estimate for mean cumulative event duration allowing for recurrent events
Wang & Quartey [56]	2013	E	Emerging (single)	No	Semi-parametric estimate for mean cumulative event duration allowing for recurrent events
Berry [57]	1989	DMP	Prespecified	Yes	Bayesian approach to estimate the posterior probability that event rate or incidence rate (incorporating exposure time) is greater in the treatment group compared to control group
French, Thomas & Wang [58]	2012	DMP	Prespecified	Yes	Bayesian logit model and a piecewise exponential models to give posterior probabilities that predefined risk difference threshold is exceeded
Yao, Zhu, Jiang & Xia [59]	2013	DMP	Prespecified	Yes	Bayesian beta-binomial model to give posterior probability that predefined risk difference threshold is exceeded
Zhu, Yao, Xia & Jiang [60]	2016^38^	DMP	Prespecified	Yes	Bayesian gamma-Poisson model to give posterior probability that predefined risk difference (incorporating exposure time) threshold is exceeded
Berry & Berry [61]	2004	DMP	Emerging (multiple)	No	Bayesian hierarchical logit model to give posterior probability that event rate greater in treatment group compared to control group
Chen, Zhao, Qin & Chen [62]	2013	DMP	Emerging (multiple)	Yes	Bayesian hierarchical logit model to give posterior probability that event rate greater in treatment group compared to control group for interim analysis
Gould [63]	2008	DMP	Emerging (multiple)	No	Bayesian approach to estimate the posterior probability that AEs in treatment group produced by a larger process than AEs in control group
Gould [64]	2013	DMP	Emerging (multiple)	No	Bayesian approach to estimate the posterior probability that AEs in treatment group produced by a larger process than AEs in control group accounting for exposure time
McEvoy, Nandy & Tiwari [65]	2013	DMP	Emerging (multiple)	No	Bayesian multivariate approach to give posterior probability of difference in event rates based on indicator functions
Xia, Ma & Carlin [66]	2011	DMP	Emerging (multiple)	No	Bayesian hierarchical logit and log-linear (incorporating exposure time) models to give posterior probability that event rate greater in treatment compared to control group

^a^*V* Visual, *HT* Hypothesis Testing, *E* Estimation, *DMP* Decision-Making Probabilities

### Summaries of methods by taxonomy

#### Visual summaries – emerging events

**Fig. 4:**
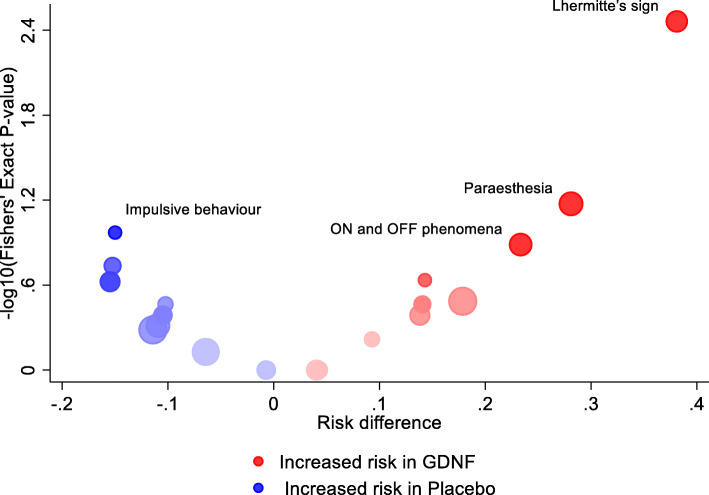
Volcano plot for adverse events experienced by at least three participants in either treatment group from Whone et al. The size of the circle represents the total number of participants with that event across treatment groups. Colour indicates direction of treatment effect. Colour saturation indicates the strength of statistical significance (calculated from whichever test the author has deemed appropriate). Circles are plotted against a measure of difference between treatment groups such as risk difference or odds ratio on the x-axis and *p*-values (with a transformation such as a log transformation) on the y-axis. Data taken from Whone et al. (2019) [67].

The review identified eight articles published between 2001 and 2018 that proposed 20 methods to visually summarise harm data, including binary AEs and, continuous laboratory (e.g. blood tests, culture data) and vital signs (e.g. temperature, blood pressure, electrocardiograms) data (Additional file 5, Table S1) [14, 25–31]. The majority of the proposed plots were designed to display summary measures of harm data (*n* = 14) and the remaining plots displayed individual participant data (*n* = 6). None of the plots required the event to be prespecified. Eight of the plots were designed to display multiple binary AEs; an example of one such plot is the volcano plot (Fig. 4) [31, 67]. The remaining plots were proposed to focus on a single event per plot, three of which proposed time-to-event plots and nine proposed plots to analyse emerging, individual, continuous harm outcomes such as laboratory or vital signs data. These plots can aid the identification of any treatment effects and identify outlying observations for further evaluation.

#### Hypothesis tests - prespecified outcomes

Five articles published between 2000 and 2012 present seven methods to analyse prespecified harm outcomes under a hypothesis-testing framework (Additional file 5, Table S2) [32–36]. Six of these methods were specifically designed and promoted for sequentially monitoring prespecified harm outcomes. Two of the methods incorporated an alpha-spending function (as originally proposed for efficacy outcomes) [22], two performed likelihood ratio tests, one used conditional power to monitor the futility of establishing safety and one proposed an arbitrary reduction in the traditional significance threshold when monitoring a harm outcome [32–34, 36]. In addition, one method proposed a non-inferiority approach for the final analysis of a prespecified harm outcome [35].

#### Hypothesis tests - emerging

Six articles published between 1990 and 2014 suggest nine methods to perform hypothesis tests to analyse emerging AE data (Additional file 5, Table S3) [37–42]. All of the methods were designed for a final analysis with one method incorporating an alpha-spending function allowing the method to be used to monitor ongoing studies. Methods are suggested for both binary and time-to-event data with several accounting for recurrent events.

Two methods proposed a *p*-value adjustment to account for multiple hypothesis tests to reduce the false discovery rate (FDR) [41, 42]. One article proposed two likelihood ratio statistics to test for differences between treatment groups when incorporating time-to-event and recurrent event data [40]. Three articles adopted multivariate approaches to undertake global likelihood ratio tests to detect differences in the overall AE profile, where the overall profile describes multiple events that are combined for evaluation [37–39].

#### Estimation – emerging

Fifteen articles proposed 24 methods published between 1991 and 2016 for emerging events (Additional file 5, Table S4) [15, 43–56]. These estimates reflect different characteristics of harm outcomes such as point estimates for incidence or duration, measures of precision around such estimates, or estimates of the probability of occurrence of events. They rely on subjective comparisons of distributional differences to identify treatment effects.

Point estimates such as the risk difference, risk ratio and odds ratio to compare treatment groups with corresponding confidence intervals (CIs) such as the binomial exact CI (also known as the Clopper-Pearson CI) are a simple approaches for AE analysis [4, 45]. Three articles proposed alternative means to estimate CIs [44, 50, 52].

Eight articles provided methods to calculate estimates that take into account AE characteristics, such as recurrent events, exposure-time, time-to-event information, and duration, which can help develop a profile of overall AE burden [15, 43, 46–48, 51, 53, 55, 56]. For example, methods such as the mean cumulative function, mean cumulative duration or parametric survival models estimating hazard ratios. Several of these methods incorporated plots that can highlight when differences between treatment groups start to emerge, which would otherwise be masked by single point estimates.

A Bayesian approach was developed to estimate the probability of experiencing different severity grades of each AE, accounting for the AE structure of events within body systems [49]. One article developed a score to indicate if continuous outcomes such as laboratory values were within normal reference ranges and to flag abnormalities [54].

#### Decision making probabilities – prespecified outcomes

Four articles suggested five Bayesian approaches to monitor prespecified harm outcomes (Additional file 5, Table S5) [57–60]. The first paper was published in 1989 but no further research was published in this area until 2012, the last paper was published in 2016. Each of the methods incorporates prior knowledge through a Bayesian framework, outputting posterior probabilities that can be used to guide the decision whether to continue with the study based on the harm outcome.

Each of the methods was designed for use in interim analyses to monitor ongoing studies but could be used for the final analysis without modification. They could be implemented for continuous monitoring (i.e. after each observed event) or in a group sequential manner after several events have occurred. These methods require a prespecified event, an assumption about the prior distribution of this event, a ‘tolerable risk difference’ and an ‘upper threshold probability’ to be set at the outset of the trial [59]. At each analysis, the probability that the ‘tolerable risk difference’ threshold is crossed is calculated and if the predetermined ‘probability threshold’ is crossed then the data indicate a predefined unacceptable harmful effect.

#### Decision making probabilities – emerging outcomes

Six articles published between 2004 and 2013 proposed eight Bayesian methods to analyse the body of emerging AE data (Additional file 5, Table S6) [61–66]. Each of the methods utilise a Bayesian framework to borrow strength from medically similar events. Berry and Berry were the first, proposing a Bayesian three-level random effects model [61]. The method allows AEs within the same body system to be more alike and information can be borrowed both within and across systems. For example, within a body system a large difference for an event amongst events with much smaller differences will be shrunk toward zero. This work was extended to incorporate person-time adjusted incidence rates using a Poisson model and to allow sequential monitoring [62, 66]. Two alternative approaches were also developed following similar principles. The output from all these models is the posterior probability that the relative measure does not equal zero or that the AE rate is greater on treatment than control.

## Discussion

In our previous work we found evidence for sub-optimal analysis practice for AE data in RCTs [4]. In this review, we set out to identify statistical methods that had been specifically developed or adapted for use in RCTs and had therefore had given full consideration to the nuances of harm data building on the recent work of Wang et al. and Zink et al. [14, 68] The aim being to improve awareness of appropriate methods. We found that despite the lack of use, there are many suitable and differing methods to undertake more sophisticated AE analysis. Some methods have been available since 1989 but most have been published since 2004. Based on our earlier work, personal experience and low citations of these articles, the uptake of these approaches appears to be minimal. The reasons for low uptake have been explored in detail in a survey of clinical trial statisticians from both academia and industry, and whilst participants indicated a moderate level of awareness of the methods summarised in this review, uptake was confirmed to be low, with a unanimous call from participants for guidance on appropriate methods for AE analysis and training to support change [69].

Issues of multiple testing, insufficient power and complex data structures are sometimes used to defend the continued practice of simple analysis approaches for AE data. For example, harm outcomes are often accompanied with additional information such as the number of occurrences, severity, timing and duration that need to be taken into consideration. However, the predominant practice is to reduce this information to simple binary counts [4, 19, 70–72]. We believe these challenges do not justify the prevalent use of simplistic analysis approaches for AE analysis.

Under the frequentist paradigm, performing multiple hypothesis tests increases the likelihood of incorrectly flagging an event due to a chance imbalance. However, when analysing harm outcomes multiple hypothesis tests can be considered less problematic than for efficacy outcomes, if incorrectly flagging an event simply means that it undergoes closer monitoring in ongoing or future trials [73]. This is supported by the recently updated New England Journal of Medicine statistical guidelines to authors that state, “*Because information contained in the safety endpoints may signal problems within specific organ classes, the editors believe that the type I error rates larger than 0.05 are acceptable*”.

Multiplicity is also not typically an issue for multivariate approaches that aim to identify global differences. Whilst these methods can be used to flag signals for differences in the overall harm profile and can help identify any differences in patient burden, a global approach to harm analysis could mask important differences at the event level. Therefore, such approaches should be considered in addition to more specific event-based analysis.

Whilst failure to consider the consequences of a lack of power can lead to inappropriate conclusions that a treatment is ‘safe’, prespecified analysis plans for prespecified events of interest would prevent post-hoc, data-driven, hypotheses testing. Nevertheless, most AE analysis is undertaken without a clear objective. Well-defined objectives setting out the purpose of the AE analysis to be undertaken for both prespecified and emerging events could help improve practice.

Visual summaries, estimation and decision-making probability methods identified in this review, are typically less obviously affected by issues of power and multiplicity since their purpose is not to undertake formal hypothesis testing to detect a statistically significant difference at a specified level of significance or power. Instead they provide a multitude of useful, alternative ways to analyse AE data where the focus is more in the frame of detecting signals for ADRs. For example, a well-designed graphic can be an effective way to communicate complex AE data to a range of audiences and help to identify signals for potential ADRs from the body of emerging AE data [27]. Similarly, estimation methods provide a means to identify distributional differences in the AE profile between treatment groups and can incorporate information on, for example, time of occurrence or recurrent events, which is often ignored in AE analysis. However, both approaches rely on visual inspections and subjective opinions regarding a decision whether to flag a signal for potential ADRs. As such, they both provide a useful means to support AE analysis but consideration of use in combination with more objective means such as statistical tests or Bayesian decision-making methods, which provide clear output for interpretation to flag differences between treatment groups, might be appropriate.

Existing knowledge on the harm profile of a drug can be used to prespecify known harm outcomes for monitoring and using an appropriate Bayesian decision-making method allows formal incorporation of existing information. Such analyses can provide evidence to aid decisions about the conduct of ongoing trials or future trials based on the emerging harm profile. Incorporating prior and/or accumulating knowledge into ongoing analyses in this way ensures an efficient use of the existing evidence allowing a cumulative assessment of harm, which is especially valuable in the context of rare events. Like the hypothesis test approaches, output can be used to objectively make decisions about whether to flag events as potential ADRs but do not suffer to the same extent with issues of insufficient power or multiplicity [74–76]. However, such methods are reliant on the prior information incorporated so sensitivity of the results to the prior assumptions should be explored and careful consideration of the appropriateness of the source of prior knowledge and its applicability is needed [77].

The most appropriate method for analysis will depend on whether events have been prespecified or are emerging and the aims of the analysis. Statistical analysis strategies could be prespecified at the outset of a trial for both prespecified and emerging events as we would for efficacy outcomes and any post-hoc exploratory analysis should be clearly identified with justification [9]. There are a multitude of specialist methods for the analysis of AEs and there is no one correct approach, rather a combination of approaches should be considered. An unwavering reliance on tables of frequencies and percentages is not necessary given the alternative methods that exist, and we urge statisticians and trialists to explore the use of more specialist analysis methods for AE data from RCTs.

We have not examined the performance of the individual methods included in this review, so we cannot make quantitative comparisons and as such have avoided making recommendations of specific methods to use. We acknowledge that single reviewer screening could have resulted in missing articles and that single reviewer data extraction could result in incorrect classifications. However, both the scoping and ongoing nature of the search and ongoing discussions between the authors regarding each article would have kept any bias to an absolute minimum. How harms are defined, trial procedures such as spontaneous versus active collection of data and coding practices are all important considerations when assessing the harm profile of an intervention. With any method it is important to remain mindful of the implications of differing practices both within and between trials when making conclusions. We have focused on original methods and the original application of existing methods for the analysis of harm outcomes. We have not searched for specific refinements of these methods and as such these would not be included in the review unless identified in the original search. Many methods that could be applied to harm analysis have not been specifically proposed for such analysis and as such are not included in this review. In this review, attention was restricted to methods specifically designed or adapted for harm outcomes to gain a better understanding on what has been done to prevent duplication in future work and to flag unknown or underutilised methods. In addition, many of the methods included in this review could be used for outcomes other than harms. There are also many methods that have been proposed for harm analysis in RCTs that were not included as they did not meet our eligibility criteria. This includes methods such as those proposed by Gould and Wang or Ball, which are both designed to be used in the RCT setting but fail to utilise a control group, combining treatment arms in an effort to preserve blinding [78, 79]. Whilst these methods have merit and offer alternative, objective ways to flag potential harms they are excluded from this review as interest lies in those methods that utilise the control group to enhance inference. This review builds on existing work to provide a comprehensive overview and audit of statistical methods available to analyse harm outcomes in clinical trials [25, 27].

## Conclusions

There are many challenges associated with assessing and analysing AE data in clinical trials. This review revealed that there are a broad range of suitable methods available to overcome some of these challenges but evidence of application in clinical trials analysis is limited. Coupled with the knowledge of barriers to implementation of such methods, development of strategies to support change are needed, thus ultimately improving analysis of harm outcomes in RCTs.

## Supplementary Information

**Additional file 1:.** Search terms by database. Full details of the search terms used to perform search.

**Additional file 2:.** Data extraction sheet. Standardised pre-piloted data extraction form.

**Additional file 3:.** Reference list of excluded articles. Reference list of articles excluded after full text review.

**Additional file 4: Figure S1.** Articles ranked according to ease of comprehension/implementation by the taxonomy of methods for adverse event (AE) analysis. Figure displaying individual articles ranked according to ease of comprehension/implementation according to the developed taxonomy.

**Additional file 5: **Tables summarising each method by taxonomy classification and type of event suitable for. **Tables S1-S6.** provide details of each method by taxonomy group and type of event suitable for.

## Data Availability

All data generated and analysed during this study either are included in this article and its supplementary information files and/or are available from the corresponding author on reasonable request.

## Abbreviations

AEs: Adverse events
ADRs: Adverse drug reactions
CI: Confidence interval
CONSORT: Consolidated Standards of Reporting Trials
FDR: False discovery rate
PRISMA: Preferred Reporting Items for Systematic Reviews and Meta-Analyses
PROSPERO: The International Prospective Register of Systematic Reviews
RCTs: Randomised controlled trials
SPERT: Safety Planning, Evaluation and Reporting Team

## Footnotes

1 An adverse event is defined as ‘any untoward medical occurrence that may present during treatment with a pharmaceutical product but which does not necessarily have a causal relationship with this treatment’. An adverse drug reaction (ADR) is defined as ‘a response to a drug which is noxious and unintended …’ where a causal relationship is ‘at least a reasonable possibility’.
