# Supplementary material for: Statistical methods for the analysis of adverse event data in randomised controlled trials: a scoping review and taxonomy
Source: BMC Med Res Methodol. 2020 Nov 30;20:288. doi: 10.1186/s12874-020-01167-9 (PMC7708917; doi:10.1186/s12874-020-01167-9)
Supplement: Supplementary file 1 — Additional file 1:. Search terms by database. Full details of the search terms used to perform search. [file 12874_2020_1167_MOESM1_ESM.docx]

Additional file 1 – Search terms by database

**Medline via Ovid**

1. Models, Statistical/

2. Models, Theoretical/

3. Biostatistics/

4. Statistics, Nonparametric/

5. Statistics as Topic/

6. Bayes Theorem/

7. Biometry/

8. Statistical Model*.mp. [mp=title, abstract, original title, name of substance word, subject heading word, keyword heading word, protocol supplementary concept word, rare disease supplementary concept word, unique identifier, synonyms]

9. Statistical Method*.mp. [mp=title, abstract, original title, name of substance word, subject heading word, keyword heading word, protocol supplementary concept word, rare disease supplementary concept word, unique identifier, synonyms]

10. Bayes* Model*.mp. [mp=title, abstract, original title, name of substance word, subject heading word, keyword heading word, protocol supplementary concept word, rare disease supplementary concept word, unique identifier, synonyms]

11. Bayes* Theor*.mp. [mp=title, abstract, original title, name of substance word, subject heading word, keyword heading word, protocol supplementary concept word, rare disease supplementary concept word, unique identifier, synonyms]

12. Bayes* Method*.mp. [mp=title, abstract, original title, name of substance word, subject heading word, keyword heading word, protocol supplementary concept word, rare disease supplementary concept word, unique identifier, synonyms]

13. 1 or 2 or 3 or 4 or 5 or 6 or 7 or 8 or 9 or 10 or 11 or 12

14. exp "Drug-Related Side Effects and Adverse Reactions"/

15. (Side effect* adj5 (monitor* or analys* or flag* or signal* or detect* or evaluat* or screen* or assess* or identif*)).mp. [mp=title, abstract, original title, name of substance word, subject heading word, keyword heading word, protocol supplementary concept word, rare disease supplementary concept word, unique identifier, synonyms]

16. ((adverse or undesirable or harm* or serious or toxic) adj3 (effect* or reaction* or event* or outcome* or experience*) adj5 (monitor* or analys* or flag* or signal* or detect* or evaluat* or screen* or assess* or identif*)).mp. [mp=title, abstract, original title, name of substance word, subject heading word, keyword heading word, protocol supplementary concept word, rare disease supplementary concept word, unique identifier, synonyms]

17. ((toxicity or complication* or noxious or tolerability) adj5 (monitor* or analys* or flag* or signal* or detect* or evaluat* or screen* or assess* or identif*)).mp. [mp=title, abstract, original title, name of substance word, subject heading word, keyword heading word, protocol supplementary concept word, rare disease supplementary concept word, unique identifier, synonyms]

18. ((AE or SAE or ADR) adj5 (monitor* or analys* or flag* or signal* or detect* or evaluat* or screen* or assess* or identif*)).mp. [mp=title, abstract, original title, name of substance word, subject heading word, keyword heading word, protocol supplementary concept word, rare disease supplementary concept word, unique identifier, synonyms]

19. (Safety adj5 (monitor* or analys* or flag* or signal* or detect* or evaluat* or screen* or assess* or identif*)).mp. [mp=title, abstract, original title, name of substance word, subject heading word, keyword heading word, protocol supplementary concept word, rare disease supplementary concept word, unique identifier, synonyms]

20. 14 or 15 or 16 or 17 or 18 or 19

21. exp Clinical Trials as Topic/

22. Clinical trial*.mp. [mp=title, abstract, original title, name of substance word, subject heading word, keyword heading word, protocol supplementary concept word, rare disease supplementary concept word, unique identifier, synonyms]

23. Clinical stud*.mp. [mp=title, abstract, original title, name of substance word, subject heading word, keyword heading word, protocol supplementary concept word, rare disease supplementary concept word, unique identifier, synonyms]

24. 21 or 22 or 23

25. 13 and 20 and 24

**EMBASE via Ovid**

1. statistical model/

2. biostatistics/

3. nonparametric test/

4. statistics/

5. bayes theorem/

6. biometry/

7. statistical analysis/

8. mathematical model/

9. Statistical Model*.mp. [mp=title, abstract, heading word, drug trade name, original title, device manufacturer, drug manufacturer, device trade name, keyword, floating subheading word]

10. Statistical Method*.mp. [mp=title, abstract, heading word, drug trade name, original title, device manufacturer, drug manufacturer, device trade name, keyword, floating subheading word]

11. Bayes* Model*.mp. [mp=title, abstract, heading word, drug trade name, original title, device manufacturer, drug manufacturer, device trade name, keyword, floating subheading word]

12. Bayes* Theor*.mp. [mp=title, abstract, heading word, drug trade name, original title, device manufacturer, drug manufacturer, device trade name, keyword, floating subheading word]

13. Bayes* Method*.mp. [mp=title, abstract, heading word, drug trade name, original title, device manufacturer, drug manufacturer, device trade name, keyword, floating subheading word]

14. 1 or 2 or 3 or 4 or 5 or 6 or 7 or 8 or 9 or 10 or 11 or 12 or 13

15. exp adverse drug reaction/

16. side effect/

17. (Side effect* adj5 (monitor* or analys* or flag* or signal* or detect* or evaluat* or screen* or assess* or identif*)).mp. [mp=title, abstract, heading word, drug trade name, original title, device manufacturer, drug manufacturer, device trade name, keyword, floating subheading word]

18. ((adverse or undesirable or harm* or serious or toxic) adj3 (effect* or reaction* or event* or outcome* or experience*) adj5 (monitor* or analys* or flag* or signal* or detect* or evaluat* or screen* or assess* or identif*)).mp. [mp=title, abstract, heading word, drug trade name, original title, device manufacturer, drug manufacturer, device trade name, keyword, floating subheading word]

19. ((toxicity or complication* or noxious or tolerability) adj5 (monitor* or analys* or flag* or signal* or detect* or evaluat* or screen* or assess* or identif*)).mp. [mp=title, abstract, heading word, drug trade name, original title, device manufacturer, drug manufacturer, device trade name, keyword, floating subheading word]

20. ((AE or SAE or ADR) adj5 (monitor* or analys* or flag* or signal* or detect* or evaluat* or screen* or assess* or identif*)).mp. [mp=title, abstract, heading word, drug trade name, original title, device manufacturer, drug manufacturer, device trade name, keyword, floating subheading word]

21. (Safety adj5 (monitor* or analys* or flag* or signal* or detect* or evaluat* or screen* or assess* or identif*)).mp. [mp=title, abstract, heading word, drug trade name, original title, device manufacturer, drug manufacturer, device trade name, keyword, floating subheading word]

22. 15 or 16 or 17 or 18 or 19 or 20 or 21

23. exp "clinical trial (topic)"/

24. Clinical trial*.mp. [mp=title, abstract, heading word, drug trade name, original title, device manufacturer, drug manufacturer, device trade name, keyword, floating subheading word]

25. Clinical stud*.mp. [mp=title, abstract, heading word, drug trade name, original title, device manufacturer, drug manufacturer, device trade name, keyword, floating subheading word]

26. 23 or 24 or 25

27. 14 and 22 and 26

**Web of Science**

TOPIC: (((Statistical near/0 Model*) or (Statistical near/0 Method*) or (Bayes* near/0 Model*) or (Bayes* near/0 Theor*) or (Bayes* near/0 Method*)) and (((Side effect*) near/5 (monitor* or analys* or flag* or signal* or detect* or evaluat* or screen* or assess* or identif*)) or ((adverse or undesirable or harm* or serious or toxic) near/3 (effect* or reaction* or event* or outcome* or experience*) near/5 (monitor* or analys* or flag* or signal* or detect* or evaluat* or screen* or assess* or identif*)) or ((toxicity or complication* or noxious or tolerability) near/5 (monitor* or analys* or flag* or signal* or detect* or evaluat* or screen* or assess* or identif*)) or ((AE or SAE or ADR) near/5 (monitor* or analys* or flag* or signal* or detect* or evaluat* or screen* or assess* or identif*)) or ((Safety) near/5 (monitor* or analys* or flag* or signal* or detect* or evaluat* or screen* or assess* or identif*))) and ((Clinical near/0 trial*) or (Clinical near/0 stud*)))

**Scopus**

( ( TITLE-ABS-KEY ( statistical W/0 model* ) ) OR ( TITLE-ABS-KEY ( statistical W/0 method* ) ) OR ( TITLE-ABS-KEY ( bayes* W/0 model* ) ) OR ( TITLE-ABS-KEY ( bayes* W/0 theor* ) ) OR ( TITLE-ABS-KEY ( bayes* W/0 method* ) ) ) AND ( ( TITLE-ABS-KEY ( "Side effect*" W/5 ( ( monitor* ) OR ( analys* ) OR ( flag* ) OR ( signal* ) OR ( detect* ) OR ( evaluat* ) OR ( screen* ) OR ( assess* ) OR ( identif* ) ) ) ) OR ( TITLE-ABS-KEY ( ( adverse OR undesirable OR harm* OR serious OR toxic ) W/3 ( effect* OR reaction* OR event* OR outcome* OR experience* ) W/5 ( monitor* OR analys* OR flag* OR signal* OR detect* OR evaluat* OR screen* OR assess* OR identif* ) ) ) OR ( TITLE-ABS-KEY ( ( toxicity OR complication* OR noxious OR tolerability ) W/5 ( monitor* OR analys* OR flag* OR signal* OR detect* OR evaluat* OR screen* OR assess* OR identif* ) ) ) OR ( TITLE-ABS-KEY ( ( ae OR sae OR adr ) W/5 ( monitor* OR analys* OR flag* OR signal* OR detect* OR evaluat* OR screen* OR assess* OR identif* ) ) ) OR ( TITLE-ABS-KEY ( ( safety ) W/5 ( monitor* OR analys* OR flag* OR signal* OR detect* OR evaluat* OR screen* OR assess* OR identif* ) ) ) ) AND ( ( TITLE-ABS-KEY ( clinical W/0 trial* ) ) OR ( TITLE-ABS-KEY ( clinical W/0 stud* ) ) )
