# Supplementary material for: Statistical methods for the analysis of adverse event data in randomised controlled trials: a scoping review and taxonomy
Source: BMC Med Res Methodol. 2020 Nov 30;20:288. doi: 10.1186/s12874-020-01167-9 (PMC7708917; doi:10.1186/s12874-020-01167-9)
Supplement: Supplementary file 2 — Additional file 2:. Data extraction sheet. Standardised pre-piloted data extraction form. [file 12874_2020_1167_MOESM2_ESM.docx]

Additional file 2 - Data extraction sheet
