# Supplementary material for: Statistical methods for the analysis of adverse event data in randomised controlled trials: a scoping review and taxonomy
Source: BMC Med Res Methodol. 2020 Nov 30;20:288. doi: 10.1186/s12874-020-01167-9 (PMC7708917; doi:10.1186/s12874-020-01167-9)
Supplement: Supplementary file 3 — Additional file 3:. Reference list of excluded articles. Reference list of articles excluded after full text review. [file 12874_2020_1167_MOESM3_ESM.docx]

**Additional file 3 – Reference list of excluded articles**

Cook, R.J., and Lawless. J. F. (1997). "MARGINAL ANALYSIS OF RECURRENT EVENTS AND A TERMINATING EVENT." Statistics in Medicine **16**(8): 911-924.

Aamot, H., et al. (2010). "Continuous monitoring of toxicity in clinical trials - Simulating the risk of stopping prematurely." International Journal of Clinical Pharmacology and Therapeutics **48**(7): 476-477.

Abrahamowicz, M., et al. (2012). "Comparison of alternative models for linking drug exposure with adverse effects." Statistics in Medicine **31**(11-12): 1014-1030.

Achim, G., et al. (2007). "Multivariate time‐to‐event analysis of multiple adverse events of drugs in integrated analyses." Statistics in Medicine **26**(7): 1518-1531.

Alemayehu, D. (2008). "Managing safety signals in large endpoint trials." Current Drug Safety **3**(2): 86-90.

Bacallao, J. and J. Lugo (1999). "Use of prior knowledge in randomized clinical trials with a binary response variable." Biotecnologia Aplicada **16**(4): 261-263.

Bai, O., et al. (2016). "Bayesian Estimation and Testing in Random Effects Meta-Analysis of Rare Binary Adverse Events." Statistics in Biopharmaceutical Research **8**(1): 49-59.

Ball, G. (2011). "Continuous safety monitoring for randomized controlled clinical trials with blinded treatment information Part 4: One method." Contemporary Clinical Trials **32**(SUPPL. 1): S11-S17.

Ball, G. (2013). "Continuous safety monitoring for randomized controlled clinical trials with blinded treatment information." Clinical Trials **2)**: S19-S20.

Ball, G. and L. B. Piller (2011). "Continuous safety monitoring for randomized controlled clinical trials with blinded treatment information Part 2: Statistical considerations." Contemporary Clinical Trials **32**: S5-S7.

Ball, G. and M. H. Silverman (2011). "Continuous safety monitoring for randomized controlled clinical trials with blinded treatment information. Part 3: Design considerations." Contemporary Clinical Trials **32**(SUPPL. 1): S8-S10.

Ball, R., et al. (2011). "Statistical, epidemiological, and risk-assessment approaches to evaluating safety of vaccines throughout the life cycle at the Food and Drug Administration." Pediatrics **127**(SUPPL. 1): S31-S38.

Bascoul-Mollevi, C., et al. (2011). "SAE: An R package for early stopping rules in clinical trials." Computer Methods and Programs in Biomedicine **104**(2): 243-248.

Berres, M. (2009). "Statistical analysis of studies on adverse events - What can they tell us?" Epilepsia **6)**: 3.

Bolland, K., et al. (2008). "A safety monitoring procedure for a clinical drug development program, with application to the assessment of a novel COX-2 inhibitor." Journal of Biopharmaceutical Statistics **18**(4): 737-749.

Bouckaert, A. and M. Mouchart (2001). "Sure outcomes of random events: a model for clinical trials." Statistics in Medicine **20**(4): 521-543.

Bradburn, M. J., et al. (2007). "Much ado about nothing: a comparison of the performance of meta-analytical methods with rare events." Statistics in Medicine **26**(1): 53-77.

Bring, J. (1995). "Stopping a clinical trial early because of toxicity: the Bayesian approach." Controlled Clinical Trials **16**(2): 131-132.

Brookhart, M. A., et al. (2012). "Characterizing vaccine-associated risks using cubic smoothing splines." American Journal of Epidemiology **176**(10): 949-957.

Brutti, P., et al. (2011). "An extension of the single threshold design for monitoring efficacy and safety in phase II clinical trials." Statistics in Medicine **30**(14): 1648-1664.

Bryant, J. and R. Day (1995). "Incorporating toxicity considerations into the design of two-stage phase II clinical trials." Biometrics **51**(4): 1372-1383.

Cabarrou, B., et al. (2016). "How to report toxicity associated with targeted therapies?" Annals of Oncology **27**(8): 1633-1638.

Cai, T., et al. (2010). "Meta-analysis for rare events." Statistics in Medicine **29**(20): 2078-2089.

Chen, C. and K. Chaloner (2006). "A Bayesian stopping rule for a single arm study: With a case study of stem cell transplantation." Statistics in Medicine **25**(17): 2956-2966.

Chen, M., et al. (2015). "Evaluation of statistical methods for safety signal detection: A simulation study." Pharmaceutical Statistics **14**(1): 11-19.

Chen, Y., et al. (2013). "Bayesian Inference on Risk Differences: An Application to Multivariate Meta-Analysis of Adverse Events in Clinical Trials." Statistics in Biopharmaceutical Research **5**(2): 142-155.

Chen, Y. and B. J. Smith (2009). "Adaptive group sequential design for phase II clinical trials: A Bayesian decision theoretic approach." Statistics in Medicine **28**(27): 3347-3362.

Chuang-Stein, C. (1998). "Safety analysis in controlled clinical trials." Drug Information Journal **32**(4 SUPPL.): 1363S-1372S.

Coleman, J. J., et al. (2006). "Monitoring for adverse drug reactions." British Journal of Clinical Pharmacology **61**(4): 371-378.

Conaway, M. R. and G. R. Petroni (1995). "Bivariate sequential designs for phase II trials." Biometrics **51**(2): 656-664.

Conaway, M. R. and G. R. Petroni (1996). "Designs for phase II trials allowing for a trade-off between response and toxicity." Biometrics **52**(4): 1375-1386.

Cook, R. J. and V. T. Farewell (1994). "Guidelines for monitoring efficacy and toxicity responses in clinical trials." Biometrics **50**(4): 1146-1152.

Cook, R.J., and Lawless. J. F. (1997). "MARGINAL ANALYSIS OF RECURRENT EVENTS AND A TERMINATING EVENT." Statistics in Medicine **16**(8): 911-924.

Crooks, C. J., et al. (2012). "Identifying adverse events of vaccines using a bayesian method of medically guided information sharing." Drug Safety **35**(1): 61-78.

Dallas, M. J. (2008). "Accounting for interim safety monitoring of an adverse event upon termination of a clinical trial." Journal of Biopharmaceutical Statistics **18**(4): 631-638.

Davis, B. and H. Southworth (2016). "Statistical Analysis of Cumulative Serious Adverse Event Data From Development Safety Update Reports." Therapeutic Innovation and Regulatory Science **50**(2): 188-194.

Davis, S., et al. (2017). "Best practices for reporting safety data to data monitoring committees." Trials. Conference: 4th International Clinical Trials Methodology Conference , ICTMC and the 38th Annual Meeting of the Society for Clinical Trials. United Kingdom **18**(Supplement 1).

Di, J., et al. (2016). "Continuous event monitoring via a Bayesian predictive approach." Pharmaceutical Statistics **15**(2): 109-122.

Dueck, A., et al. (2016). "Comparison of statistical analysis approaches for ordinal symptom scales used for patient-reported side effects in cancer treatment clinical trials." Supportive Care in Cancer **24 (1 Supplement 1)**: S196.

Dueck, A., et al. (2017). "Statistical analysis strategies for PRO-CTCAE data in oncology clinical trials: A simulation study." Trials. Conference: 4th International Clinical Trials Methodology Conference , ICTMC and the 38th Annual Meeting of the Society for Clinical Trials. United Kingdom **18**(Supplement 1).

Duke, S. P., et al. (2017). "Quantitative Methods for Safety Monitoring of Rare Serious Adverse Events." Pharmaceutical Medicine **31**(2): 113-118.

DuMouchel, W. (2012). "Multivariate Bayesian Logistic Regression for Analysis of Clinical Study Safety Issues." Statistical Science **27**(3): 319-339.

Enas, G. G. (1991). "Making decisions about safety in clinical trials - The case for inferential statistics." Drug Information Journal **25**(3): 439-446.

Enas, G. G. and D. J. Goldstein (1995). "Defining, monitoring and combining safety information in clinical trials." Statistics in Medicine **14**(9-10): 1099-1111.

Etzioni, R. and M. S. Pepe (1994). "Monitoring of a pilot toxicity study with two adverse outcomes." Statistics in Medicine **13**(22): 2311-2321.

Feldmann, U. (1993). "Epidemiologic assessment of risks of adverse reactions associated with intermittent exposure." Biometrics **49**(2): 419-428.

Fernandes, L. L., et al. (2016). "Multivariate Markov models for the conditional probability of toxicity in phase II trials." Biometrical Journal Biometrische Zeitschrift. **58**(1): 186-205.

Fescharek, R., et al. (1998). "Monitoring and safety assessment in Phase I to III clinical trials." Developments in Biological Standardization **95**: 203-209.

Fu, H., et al. (2013). "Identifying potential adverse events dose-response relationships via Bayesian indirect and mixed treatment comparison models." Journal of Biopharmaceutical Statistics **23**(1): 26-42.

Furey, A. and R. Bechhofer (2017). "Effective graphical analyses of adverse events in DMC reports." Trials. Conference: 4th International Clinical Trials Methodology Conference , ICTMC and the 38th Annual Meeting of the Society for Clinical Trials. United Kingdom **18**(Supplement 1).

Gait, J. E., et al. (2000). "Evaluation of safety data from controlled clinical trials: The clinical principles explained." Drug Information Journal **34**(1): 273-287.

Gao, F., et al. (2007). "Non-parametric estimation for baseline hazards function and covariate effects with time-dependent covariates." Statistics in Medicine **26**(4): 857-868.

Gibbons, R. D., et al. (2010). "Post-approval drug safety surveillance." Annual Review of Public Health **31**: 419-437.

Goldberg-Alberts, R. and S. Page (2006). "Multivariate analysis of adverse events." Drug Information Journal **40**(1): 99-110.

Goldman, A. I. and P. J. Hannan (2001). "Optimal continuous sequential boundaries for monitoring toxicity in clinical trials: a restricted search algorithm." Statistics in Medicine **20**(11): 1575-1589.

Gould, A. L. (2015). Statistical Methods for Evaluating Safety in Medical Product Development, Wiley Blackwell.

Gould, A. L. (2016). "Control charts for monitoring accumulating adverse event count frequencies from single and multiple blinded trials." Statistics in Medicine **35**(30): 5561-5578.

Gould, A. L. and W. B. Wang (2017). "Monitoring potential adverse event rate differences using data from blinded trials: the canary in the coal mine." Statistics in Medicine **36**(1): 92-104.

Gruber, S. and M. J. Van der Laan (2013). "An Application of Targeted Maximum Likelihood Estimation to the Meta-Analysis of Safety Data." Biometrics **69**(1): 254-262.

Grunkemeier, G. L., et al. (2014). "Bayesian stopping guidelines for heart valve premarket approval studies." Journal of Thoracic and Cardiovascular Surgery **148**(6): 2813-2817.

Herson, J. (2015). Safety Monitoring. Statistical Methods for Evaluating Safety in Medical Product Development, Wiley Blackwell**:** 293-318.

Huang, J., et al. (2011). "Adverse event signal detection: Overall comparisons, future projections and false discoveries." Clinical Trials **8 (4)**: 475.

Huster, W. J. (1991). "Clinical trial adverse events: The case for descriptive techniques." Drug Information Journal **25**(3): 447-456.

Islam, S. S., et al. (2016). "Real time aggregate clinical safety monitoring methodology-evaluation of multiple quantitative methods." Pharmacoepidemiology and Drug Safety **25 (Supplement 3)**: 140.

Ivanova, A., et al. (2005). "Continuous toxicity monitoring in phase II trials in oncology." Biometrics **61**(2): 540-545+652.

Ivanova, A., et al. (2015). "Monitoring rules for toxicity in Phase II oncology trials." Clinical Investigation **5**(4): 373-381.

Jones, J. K. (1986). "Epidemiologic perspective on causality assessment for drug associated events." Drug Information Journal **20**(4): 413-421.

Jones, J. K. (2001). "The role of data mining technology in the identification of signals of possible adverse drug reactions: Value and limitations." Current Therapeutic Research - Clinical and Experimental **62**(9): 664-672.

Kaiser, L. D., et al. (2010). "Optimizing collection of adverse event data in cancer clinical trials supporting supplemental indications." Journal of Clinical Oncology **28**(34): 5046-5053.

Kajungu, D. K., et al. (2014). "Paediatric pharmacovigilance: Use of pharmacovigilance data mining algorithms for signal detection in a safety dataset of a paediatric clinical study conducted in seven African countries." PLoS ONE **9**(5).

Kashiwabara, K., et al. (2014). "A Bayesian Stopping Rule for Sequential Monitoring of Serious Adverse Events." Therapeutic Innovation and Regulatory Science **48**(4): 444-452.

Kieser, M. (2016). "Statistical methods for the analysis of adverse event data." Pharmaceutical Statistics **15**(4): 290-291.

Kim, K. M. (2010). "Interim reports for data monitoring committee vs clinical study reports for regulatory authorities." Clinical Trials **7 (4)**: 443.

Kim, K. M. (2011). "Interim reports for data monitoring committee review vs final reports for regulatory filing." Trials. Conference: Clinical Trials Methodology Conference **12**(SUPPL. 1).

Korn, E. L., et al. (1993). "Stopping a clinical trial very early because of toxicity: summarizing the evidence." Controlled Clinical Trials **14**(4): 286-295.

Kramar, A. and C. Bascoul-Mollevi (2009). "Early Stopping Rules in Clinical Trials Based on Sequential Monitoring of Serious Adverse Events." Medical Decision Making **29**(3): 343-350.

Lachin, J. M. (1981). "Sequential clinical trials for normal variates using interval composite hypotheses." Biometrics **37**(1): 87-101.

Lan, K. K. and L. Friedman (1986). "Monitoring boundaries for adverse effects in long-term clinical trials." Controlled Clinical Trials **7**(1): 1-7.

Lane, P. W. (2013). "Meta-analysis of incidence of rare events." Statistical Methods in Medical Research **22**(2): 117-132.

Lin, X., et al. (2012). "Truncated robust distance for clinical laboratory safety data monitoring and assessment." Journal of Biopharmaceutical Statistics **22**(6): 1174-1192.

Liu, J., et al. (2015). "Evaluation of vaccine seroresponse rates and adverse event rates through Bayesian and frequentist methods." Human vaccines & Immunotherapeutics **11**(6): 1557-1563.

Liu, M., et al. (2012). "Large-scale prediction of adverse drug reactions using chemical, biological, and phenotypic properties of drugs." Journal of the American Medical Informatics Association **19**(e1): e28-35.

Ma, H., et al. (2015). "Statistical Considerations on the Evaluation of Imbalances of Adverse Events in Randomized Clinical Trials." Therapeutic Innovation and Regulatory Science **49**(6): 957-965.

Maansson, R., et al. (2018). "Modeling excess zeroes in an integrated analysis of vaccine safety." Human Vaccines and Immunotherapeutics: 1-4.

McEvoy, B. W. and R. C. Tiwari (2012). "Discussion of "Multivariate Bayesian Logistic Regression for Analysis of Clinical Trial Safety Issues" by W. DuMouchel." Statistical Science **27**(3): 340-343.

Merz, M., et al. (2014). "Methodology to Assess Clinical Liver Safety Data." Drug Safety **37**(1): 33-45.

O'Connell, M. (2006). "Drug safety, the U.S. food and drug administration and statistical data mining enabling data mining, statistical analysis and graphical reporting to run smoothly." Scientific Computing **23**(7): 32-33.

Odani, M., et al. (2017). "A Bayesian meta-analytic approach for safety signal detection in randomized clinical trials." Clinical Trials **14**(2): 192-200.

O'Neill, R. T. (1987). "Statistical analyses of adverse event data from clinical trials. Special emphasis on serious events." Drug Information Journal **21**(1): 9-20.

Peace, K. E. (1987). "Design, monitoring, and analysis issues relative to adverse events." Drug Information Journal **21**(1): 21-28.

Pedroza, C., et al. (2016). "Advantages of Bayesian monitoring methods in deciding whether and when to stop a clinical trial: an example of a neonatal cooling trial." Trials [Electronic Resource] **17**(1): 335.

Pere, J. C., et al. (1986). "Computerized comparison of six adverse drug reaction assessment procedures." Clinical Pharmacology and Therapeutics **40**(4): 451-461.

Phillips, R. and V. Cornelius (2017). "Overview of statistical methods to monitor harms during the conduct of a randomised controlled trial." Trials. Conference: 4th International Clinical Trials Methodology Conference , ICTMC and the 38th Annual Meeting of the Society for Clinical Trials. United Kingdom **18**(Supplement 1).

Price, K. L., et al. (2014). "Bayesian methods for design and analysis of safety trials." Pharmaceutical Statistics **13**(1): 13-24.

Prieto-Merino, D. and S. J. W. Evans (2009). "Effects of different groupings of drug adverse events in the detection of signals using Bayesian analyses." Pharmacoepidemiology and Drug Safety (PDS) **18 (S1)**: S211.

Prieto-Merino, D. and S. J. W. Evans (2009). "Using different types of priors in a Bayesian Hierarchical Model for the analysis of drug adverse events." Pharmacoepidemiology and Drug Safety (PDS) **18 (S1)**: S190.

Proctor, T. and M. Schumacher (2016). "Analysing adverse events by time-to-event models: the CLEOPATRA study." Pharmaceutical Statistics **15**(4): 306-314.

Propert, K. J. and J. R. Anderson (1988). "Assessing the effect of toxicity on prognosis: Methods of analysis and interpretation." Journal of Clinical Oncology **6**(5): 868-870.

Rose, C. E., et al. (2006). "On the use of zero-inflated and hurdle models for modeling vaccine adverse event count data." Journal of Biopharmaceutical Statistics **16**(4): 463-481.

Scholtens, D. and R. A. Betensky (2006). "A computationally simple bivariate survival estimator for efficacy and safety." Lifetime Data Analysis **12**(3): 365-387.

Scott, J. A., et al. (2011). "BayesWeb: A USER-FRIENDLY PLATFORM FOR EXPLORATORY BAYESIAN ANALYSIS OF SAFETY SIGNALS FROM SMALL CLINICAL TRIALS." Journal of Biopharmaceutical Statistics **21**(5): 1030-1041.

Shapiro, D. R. and T. J. Cook (1994). "Analysis of long-term adverse experience data using the Weibull model." Drug Information Journal **28**(2): 541-552.

Shen, L. Z., et al. (2008). "A Bayesian approach to utilizing prior data in new drug development." Journal of Biopharmaceutical Statistics **18**(2): 227-243.

Sogliero-Gilbert, G. et al. (1986). "A Procedure for the Simplification and Assessment of Lab Parameters in Clinical Trials." Therapeutic Innovation & Regulatory Science **20**(3): 279 - 296.

Song, G. and A. Ivanova (2015). "Enrollment and Stopping Rules for Managing Toxicity Requiring Long Follow-Up in Phase II Oncology Trials." Journal of Biopharmaceutical Statistics **25**(6): 1206-1214.

Southworth, H. and J. E. Heffernan (2012). "Extreme value modelling of laboratory safety data from clinical studies." Pharmaceutical Statistics **11**(5): 361-366.

Southworth, H. and M. O'Connell (2009). "Data mining and statistically guided clinical review of adverse event data in clinical trials." Journal of Biopharmaceutical Statistics **19**(5): 803-817.

Sugar, E., et al. (2013). "Graphical methods for monitoring clinical trial data." Clinical Trials **2)**: S34.

Sutton, A. J., et al. (2002). "Meta-analysis of rare and adverse event data." Expert Review of Pharmacoeconomics and Outcomes Research **2**(4): 367-379.

Teramukai, S., et al. (2015). "An extension of Bayesian predictive sample size selection designs for monitoring efficacy and safety." Statistics in Medicine **34**(22): 3029-3039.

Thall, P. F., et al. (1996). "New statistical strategy for monitoring safety and efficacy in single-arm clinical trials." Journal of Clinical Oncology **14**(1): 296-303.

Thall, P. F., et al. (2005). "Monitoring event times in early phase clinical trials: Some practical issues." Clinical Trials **2**(6): 467-478.

Thanarajasingam, G., et al. (2016). "Longitudinal adverse event assessment in oncology clinical trials: the Toxicity over Time (ToxT) analysis of Alliance trials NCCTG N9741 and 979254." Lancet Oncology **17**(5): 663-670.

Thanarajasingam, G., et al. (2016). "Beyond maximum grade: A novel method to assess toxicity over time in clinical trials of targeted therapy in lymphoma." Journal of Clinical Oncology. Conference **34**(Supplement 15).

Thanarajasingam, G., et al. (2017). "Beyond maximum grade: A novel, longitudinal toxicity over time (TOXT) adverse event analysis of lenalidomide in follicular lymphoma in CALGB 50401 (Alliance)." Hematological Oncology **35 (Supplement 2)**: 213-215.

Todd, S. (2003). "An adaptive approach to implementing bivariate group sequential clinical trial designs." Journal of Biopharmaceutical Statistics **13**(4): 605-619.

Tohme, M., et al. (2010). "A multiclass multivariate group comparison test: Application to drug safety." Conference proceedings : .. Annual International Conference of the IEEE Engineering in Medicine and Biology Society. IEEE Engineering in Medicine and Biology Society. Conference.: 4711-4714.

Tuber‐Bitter, P., et al. (1995). "COMPARING THE BIVARIATE EFFECTS OF TOXICITY AND EFFICACY OF TREATMENTS." Statistics in Medicine **14**(9): 1129-1141.

Walker, A. M. and M. Kulldorff (2010). "Sequential analysis for safety surveillance." Pharmacoepidemiology and Drug Safety **1)**: S88-S89.

Wang, Y., et al. (2001). "Evaluate multiple adverse events in crossover design bioequivalence clinical trials." Acta Pharmacologica Sinica **22**(2): 187-192.

Weaver, J., et al. (2016). "Strategies on Using Prior Information When Assessing Adverse Events." Statistics in Biopharmaceutical Research **8**(1): 106-115.

Xia, H. A. and Q. Jiang (2014). "Statistical Evaluation of Drug Safety Data." Therapeutic Innovation & Regulatory Science **48**(1): 109-120.

Yang, G., et al. (2016). "Meta-analysis framework for exact inferences with application to the analysis of rare events." Biometrics **72**(4): 1378-1386.

Yu, J., et al. (2016). "Group sequential control of overall toxicity incidents in clinical trials - Non-Bayesian and Bayesian approaches." Statistical Methods in Medical Research **25**(1): 64-80.

Yuan, Y. N., et al. (2012). "Visual approaches to evaluate adverse event data in clinical trials. [Chinese]." Chinese Journal of New Drugs **21**(6): 647-653.

Zingmark, P. H., et al. (2005). "Modelling a spontaneously reported side effect by use of a Markov mixed-effects model." Journal of Pharmacokinetics and Pharmacodynamics **32**(2): 261-281.
