# Supplementary material for: Statistical methods for the analysis of adverse event data in randomised controlled trials: a scoping review and taxonomy
Source: BMC Med Res Methodol. 2020 Nov 30;20:288. doi: 10.1186/s12874-020-01167-9 (PMC7708917; doi:10.1186/s12874-020-01167-9)
Supplement: Supplementary file 4 — Additional file 4: Figure S1. Articles ranked according to ease of comprehension/implementation by the taxonomy of methods for adverse event (AE) analysis. Figure displaying individual articles ranked according to ease of comprehension/implementation according to the developed taxonomy. [file 12874_2020_1167_MOESM4_ESM.pdf]

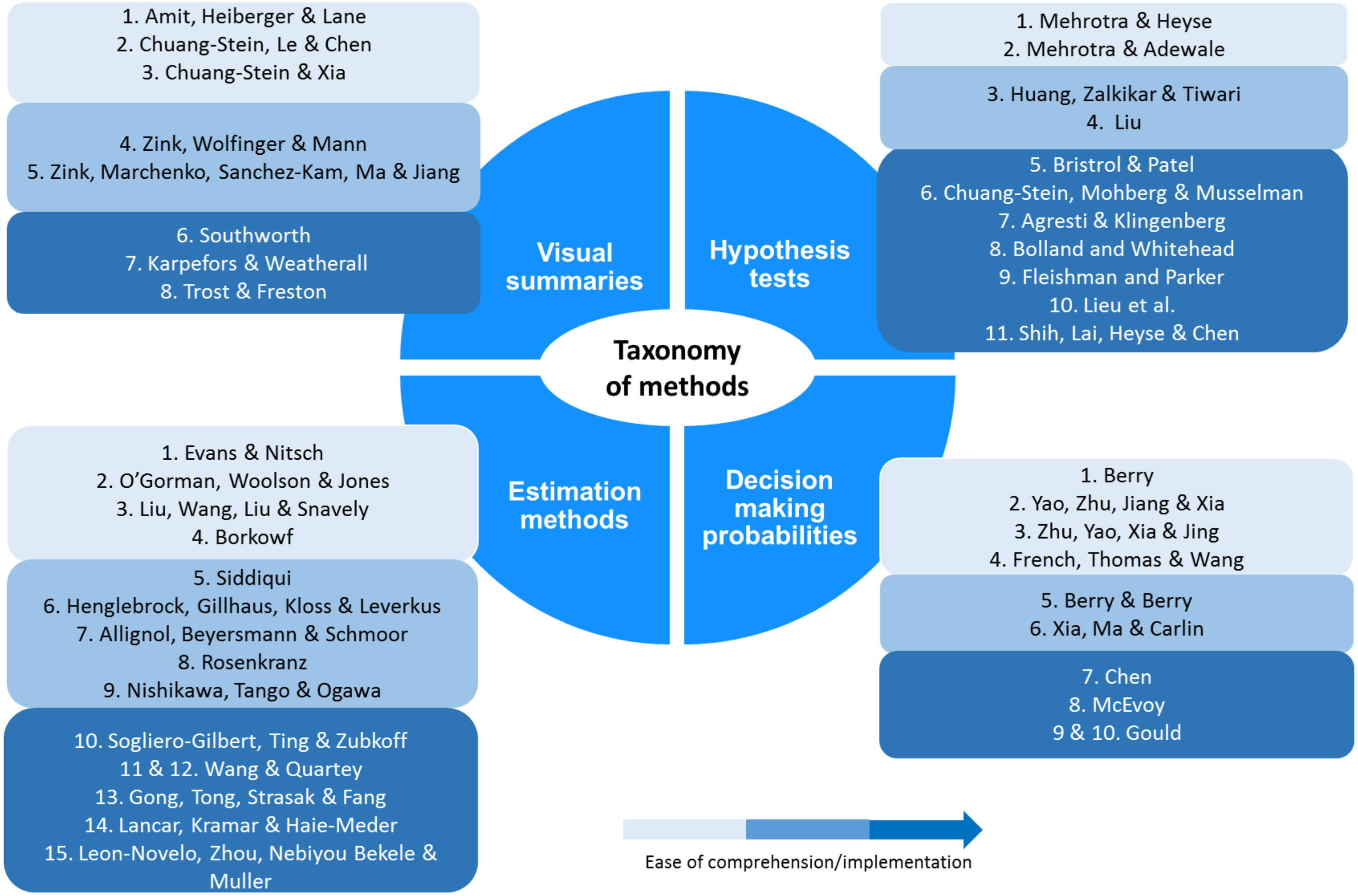

Figure S1: Articles ranked according to ease of comprehension/implementation by the taxonomy of methods for adverse event (AE) analysis
