# Supplementary material for: Statistical methods for the analysis of adverse event data in randomised controlled trials: a scoping review and taxonomy
Source: BMC Med Res Methodol. 2020 Nov 30;20:288. doi: 10.1186/s12874-020-01167-9 (PMC7708917; doi:10.1186/s12874-020-01167-9)
Supplement: Supplementary file 5 — Additional file 5: Tables summarising each method by taxonomy classification and type of event suitable for. Tables S1-S6. provide details of each method by taxonomy group and type of event suitable for. [file 12874_2020_1167_MOESM5_ESM.pdf]

Additional file 5 –Tables summarising each method by taxonomy classification and type of event suitable for

Table S1: Summary of visual approaches to summarise AE data in phase II/III RCTs

Outcome	Data type	Plot	Reference	Brief Description
**Emerging adverse events (multiple)**	Binary	Volcano	Zink, Wolfinger & Mann 2013. Xia 2011 first proposed this method for systematic reviews but was not eligible for inclusion in this review.[1 2]	Summarises and compares the incidence of each AE reported by treatment group
	Binary	Dot	Amit, Heiberger & Lane, 2008. Cooper, 2008 also proposed but for pooled trials therefore not eligible for inclusion in this review.[3 4]	Provides an absolute and relative measure compared across treatment group for each AE reported
	Time-to-event	Tendril	Karpefors & Weatherall, 2018.[5]	Provides a summary of time-to-event data by treatment group for each AE reported
	Binary	Heat map	Zink, Marchenko, Sanchez-Kam, Ma & Jiang[6]	Visualises treatment effects for each AE reported
	Binary	Bar chart	Chuang-Stein & Xia, 2013.[7]	Displays frequency of events
	Binary	Venn diagram	Chuang-Stein & Xia, 2013.[7]	Presents frequencies highlighting the prevalence of overlapping events
	Binary	Two-by-two frequencies	Chuang-Stein, Le & Chen, 2001.[8]	Displays two-by-two frequencies graphically
	Binary	Forest plot	Chuang-Stein & Xia, 2013. Refers to Lewis and Clarke, 2001 which is not eligible in own right as it's not specific to AEs.[7 9]	Plots a relative measure compared across treatment groups for each AE reported within a group such as body system
**Emerging adverse events (single)**	Time-to-event	Kaplan-Meier	Amit, Heiberger & Lane, 2008.[3]	Summarises time-to-event data highlighting absolute differences over time
	Time-to-event	Hazard function	Amit, Heiberger & Lane, 2008.[3]	Summarises time-to-event data highlighting the time at which differences emerge
	Time-to-event	Risk over time	Chuang-Stein & Xia, 2013.[7]	Summarises incidence of an event over time
**(Emerging)**	Continuous	Cumulative frequency plots/empirical cumulative distribution function	Amit, Heiberger & Lane, 2008.[3]	Provides a summary of the distribution e.g. change for individual participants over time

**Laboratory & Vital Signs (single or multiple)**		Boxplots	Amit, Heiberger & Lane, 2008. Cooper, 2008 also proposed but for pooled trials therefore not eligible for inclusion in this review.[3 4]	Provides a summary of the distribution e.g. change at specific time points
		Line graphs	Amit, Heiberger & Lane, 2008.[3]	Provides a summary of change at specific time points
		Histograms	Chuang-Stein, Le & Chen, 2001.[8]	Provides a summary of the distribution e.g. change at specific time points
		Scatter plots	Amit, Heiberger & Lane, 2008. Cooper, 2008 also proposed but for pooled trials therefore not eligible for inclusion in this review.[3 4]	Provides a summary of change for individual participants over time
		Scatter plot with regression	Southworth, 2008.[10]	Provides a summary of change for individual participants over time highlighting any outliers
		Delta	Chuang-Stein, Le & Chen, 2001.[8]	Display individual participant changes
		Vector plots	Trost & Freston, 2008.[11]	Simultaneously displays individual participant changes across three laboratory values
		e-Dish	Chuang-Stein & Xia, 2013.[7]	Scatter plot of peak serum ALT and peak bilirubin levels for individual participants to identify drug induced serious hepatotoxicity

Table S2: Summary of hypothesis tests to analyse prespecified harm outcomes in phase II/III RCTs

Outcome	Data type	Model	Reference	Brief Description
**Prespecified safety outcome**	Binary	Logit	Bolland & Whitehead[12]	Alpha-spending function for sequential monitoring
	Binary, count, time-to-event or continuous	Not applicable	Fleishman & Parker[13]	Redefine significance threshold for sequential monitoring
	Binary, count, time-to-event or continuous	Not applicable		Conditional power at each interim analysis for sequential monitoring
	Time-to-event	Exponential		Alpha-spending function for sequential monitoring
	Binary or incidence rate	Binomial or Poisson	Lieu et al.[14]	Maximised sequential probability ratio test for sequential monitoring
	Binary, count - incident rate	Poisson	Shih, Lai, Heyse & Chen[15]	Sequential generalised likelihood ratio test for sequential monitoring
	Binary, count, time-to-event or continuous	Not applicable	Liu[16]	Non-inferiority test for final analysis

Table S3: Summary of hypothesis tests to analyse emerging AE data in phase II/III RCTs

Outcome	Data type	Model	Reference	Brief Description
**Emerging Adverse events (multiple)**	Binary	Not applicable	Mehrotra & Heyse[17]	P-value adjustment
	Binary	Not applicable	Mehrotra & Adewale[18]	P-value adjustment
**Emerging Adverse events (single)**	Time-to-event	Poisson	Huang, Zalkikar & Tiwari[19]	Likelihood ratio test to compare relative risk for time-to-first event
	Time-to-event	Poisson		Likelihood ratio test to compare relative risk allowing recurrent events
**Overall adverse event profile***	Binary	Multivariate - Markov chain	Bristol & Patel[20]	Multivariate likelihood ratio test with Markov chain of order one
	Binary	Multivariate - chi-squared	Chuang-Stein Mohberg & Musselman[21]	Multivariate test with chi-squared distribution
	Binary	Multinomial - logit	Agresti & Klingenberg[22]	Likelihood ratio test using a logit models to test for equality of two vectors for the marginal distributions
	Binary	Exact permutation distribution		Likelihood ratio test using the exact permutation distribution to test joint distributions
	Binary	Multinomial - logistic normal random intercept model		Likelihood ratio test using a logistic normal random intercept model to compare marginal distributions whilst modelling the joint distributions

* Where the overall adverse event profile describes multiple events that are somehow combined for evaluation.

Table S4: Summary of estimation approaches to analyse emerging AE data in phase II/III RCTs

Outcome	Data type	Estimate	Reference	Brief Description
**Emerging Adverse events (multiple)**	Ordinal	Posterior probability	Leon-Novelo, Zhou, Nebiyou Bekele & Muller[23]	Posterior probability of each grade of an AE (participant maximum grade used) allowing multiple different events per participant
**Emerging Adverse events (single)**	Binary	Frequencies & percentage	Evans & Nitsch[24]	Standard estimates for AE analysis including frequencies, percentages, risk differences and odds ratios
	Binary	Regression models	Evans & Nitsch[24]	Regression based approaches for AE analysis e.g. Poisson regression
	Binary	Confidence interval	O'Gorman, Woolson, Jones[25]	Two methods to estimate CIs for risk-difference when combing data across multiple sites
	Count	Confidence interval	Liu, Wang, Liu & Snavely[26]	Four methods to estimate CIs for exposure adjusted incident ratios
	Binary	Confidence interval	Borkowf[27]	Alternative to the Clopper-Pearson CI for proportions
	Binary	Mean cumulative function	Siddiqui[28]	Non-parametric estimate of mean cumulative number of recurrent events
	Binary	Prevalence	Lancar, Kramar & Haie-Meder[29]	Non-parametric estimate of prevalence of event allowing for recurrence
	Binary	Mean frequency function	Gong, Tong, Strasak & Fang[30]	Non-parametric estimate of mean cumulative number of recurrent events in presence of competing risks
	Binary	Mean cumulative duration	Wang & Quartey, 2012[31]	Non-parametric estimate of mean cumulative duration for recurrent events
	Binary	Mean cumulative duration	Wang & Quartey, 2013[32]	Semi-parametric estimate of mean cumulative duration and prevalence of recurrent events
	Binary	Dependence between AEs and discontinuation	Rosenkranz[33]	Three methods to estimate the level of dependence between AE and discontinuation by treatment group that corrects for any dependence in the treatment effect estimate
	Time-to-event	Hazard ratio	Henglebrock, Gillhaus, Kloss & Leverkus[34]	Two methods to estimate hazard ratio for recurrent events

	Time-to-event	Cumulative incidence function	Allignol, Beyersmann & Schmoor[35]	Two methods to estimate the probability of an event in presence of competing risks
	Time-to-event	Conditional cumulative incidence function	Nishikawa, Tango & Ogawa[36]	Probability of a recurrent event in presence of competing risks
**(Emerging) Laboratory & vital signs (multiple)**	Continuous	GENIE score	Sogliero-Gilbert, Ting, & Zubkoff[37]	Weighted linear combination of absolute normalised deviations from the reference range to indicate abnormalities

Table S5: Summary of decision making probability methods to analyse prespecified harm outcomes in phase II/III RCTs

Outcome	Data type	Model	Prior	Reference	Brief Description
**Predefined safety outcome**	Binary	Beta-Binomial	Beta	Berry[38]	Posterior probability that event rate or incidence rate (incorporating exposure time) is greater in the treatment group compared to control group
	Time-to-event	Exponential	Not specified		
	Binary	Beta-Binomial	Beta	Yao, Zhu, Jiang & Xia[39]	Beta-binomial model to give posterior probability that predefined risk difference threshold is exceeded
	Count	Gamma-Poisson	Gamma	Zhu, Yao, Xia & Jiang[40]	Gamma-Poisson model to give posterior probability that predefined risk difference (incorporating exposure time) threshold is exceeded
	Binary	Logit model	Normal	French, Thomas and Wang[41]	Logit model and a piecewise exponential model to give posterior probabilities that predefined risk difference threshold is exceeded
	Time-to-event	Piecewise exponential	Normal & Gamma		

Table S6: Summary of decision making probability methods to analyse emerging AE data in phase II/III RCTs

Outcome	Data type	Model	Prior	Reference	Brief Description
**Emerging Adverse events (multiple)**	Binary	Logit	Mixed	Berry & Berry[42]	Bayesian hierarchical logit model to give posterior probability that event rate greater in treatment compared to control group
	Binary	Logit	Normal	Xia, Ma &Carlin[43]	Bayesian hierarchical logit and log-linear (incorporating exposure time) models to give posterior probability that event rate greater in treatment compared to control group
	Count	Log (Poisson)	Mixed		
	Count	Log (Poisson)	Normal		
	Binary	Logit	Mixed	Chen[44]	Sequential method. Bayesian hierarchical logit model to give posterior probability that event rate greater in treatment compared to control group for interim analysis
	Binary	Beta-Binomial	Isling	McEvoy[45]	Multivariate approach to give posterior probability of difference in event rates based on indicator functions
	Binary	Beta-Binomial	Beta	Gould[46]	Posterior probability that AEs in treatment group produced by a larger process than AE in control group
	Count	Gamma-Poisson	Poisson	Gould[47]	Posterior probability that AEs in treatment group produced by a larger process than AE in control group accounting for exposure time

References for tables S1-S6

1. Zink RC, Wolfinger RD, Mann G. Summarizing the incidence of adverse events using volcano plots and time intervals. *Clinical Trials* 2013;10(3):398-406.
2. Xia HA, Crowe BJ, Schriver RC, et al. Planning and core analyses for periodic aggregate safety data reviews. *Clinical Trials* 2011;8(2):175-82. doi: 10.1177/1740774510395635
3. Amit O, Heiberger RM, Lane PW. Graphical approaches to the analysis of safety data from clinical trials. *Pharmaceutical Statistics* 2008;7(1):20-35.
4. Cooper AJP, Lettis S, Chapman CL, et al. Developing tools for the safety specification in risk management plans: lessons learned from a pilot project. *Pharmacoepidemiology and Drug Safety* 2008;17(5):445-54. doi: doi:10.1002/pds.1576
5. Karpefors M, Weatherall J. The Tendril Plot—a novel visual summary of the incidence, significance and temporal aspects of adverse events in clinical trials. *Journal of the American Medical Informatics Association* 2018;25(8):1069-73. doi: 10.1093/jamia/ocy016
6. Zink RC, Marchenko O, Sanchez-Kam M, et al. Sources of Safety Data and Statistical Strategies for Design and Analysis:Clinical Trials. *Therapeutic Innovation & Regulatory Science* 2018;52(2):141-58. doi: 10.1177/2168479017738980
7. Chuang-Stein C, Xia HA. The practice of pre-marketing safety assessment in drug development. *Journal of Biopharmaceutical Statistics* 2013;23(1):3-25. doi: 10.1080/10543406.2013.736805
8. Chuang-Stein C, Le V, Chen W. Recent Advancements in the Analysis and Presentation of Safety Data. *Drug Information Journal* 2001;35(2):377-97. doi: 10.1177/009286150103500207
9. Lewis S, Clarke M. Forest plots: trying to see the wood and the trees. *BMJ* 2001;322(7300):1479-80. doi: 10.1136/bmj.322.7300.1479
10. Southworth H. Detecting outliers in multivariate laboratory data. *Journal of Biopharmaceutical Statistics* 2008;18(6):1178-83.
11. Trost DC, Freston JW. Vector Analysis to Detect Hepatotoxicity Signals in Drug Development. *Therapeutic Innovation & Regulatory Science* 2008;42(1):27-34. doi: 10.1177/009286150804200106
12. Bolland K, Whitehead J. Formal approaches to safety monitoring of clinical trials in life-threatening conditions. *Statistics in Medicine* 2000;19(21):2899-917. doi: 10.1002/1097-0258(20001115)19:21<2899::AID-SIM597>3.0.CO;2-O
13. Fleishman AN, Parker RA. Stopping guidelines for harm in a study designed to establish the safety of a marketed drug. *Journal of Biopharmaceutical Statistics* 2012;22(2):338-50. doi: 10.1080/10543406.2010.536872
14. Lieu TA, Kulldorff M, Davis RL, et al. Real-time vaccine safety surveillance for the early detection of adverse events. *Medical Care* 2007;45(10 SUPPL. 2):S89-S95.
15. Shih MC, Lai TL, Heyse JF, et al. Sequential generalized likelihood ratio tests for vaccine safety evaluation. *Statistics in Medicine* 2010;29(26):2698-708.
16. Liu JP. Rethinking statistical approaches to evaluating drug safety. *Yonsei Medical Journal* 2007;48(6):895-900. doi: 10.3349/ymj.2007.48.6.895
17. Mehrotra DV, Heyse JF. Use of the false discovery rate for evaluating clinical safety data. *Statistical Methods in Medical Research* 2004;13(3):227-38.
18. Mehrotra DV, Adewale AJ. Flagging clinical adverse experiences: Reducing false discoveries without materially compromising power for detecting true signals. *Statistics in Medicine* 2012;31(18):1918-30.
19. Huang L, Zalkikar J, Tiwari R. Likelihood ratio based tests for longitudinal drug safety data. *Statistics in Medicine* 2014;33(14):2408-24. doi: 10.1002/sim.6103
20. Bristol DR, Patel HI. A Markovian model for comparing incidences of side effects. *Statistics in Medicine* 1990;9(7):803-09.

21. Chuang-Stein C, Mohberg NR, Musselman DM. Organization and analysis of safety data using a multivariate approach. *Statistics in Medicine* 1992;11(8):1075-89. doi: doi:10.1002/sim.4780110809
22. Agresti AaK, B. Multivariate tests comparing binomial probabilities, with application to safety studies for drugs. *Appl Statist* 2005;54(4):691-706.
23. Leon-Novelo LG, Zhou X, Bekele BN, et al. Assessing toxicities in a clinical trial: Bayesian inference for ordinal data nested within categories. *Biometrics* 2010;66(3):966-74.
24. Evans SJW, Nitsch D. Statistics: Analysis and Presentation of Safety Data. In: Talbot J, Aronson JK, eds. Stephens' Detection and Evaluation of Adverse Drug Reactions: Principles and Practice. Sixth Edition ed: John Wiley and Sons 2012:349-88.
25. O'Gorman TW, Woolson RF, Jones MP. A comparison of two methods of estimating a common risk difference in a stratified analysis of a multicenter clinical trial. *Controlled Clinical Trials* 1994;15(2):135-53.
26. Liu GF, Wang J, Liu K, et al. Confidence intervals for an exposure adjusted incidence rate difference with applications to clinical trials. *Statistics in Medicine* 2006;25(8):1275-86. doi: doi:10.1002/sim.2335
27. Borkowf CB. Constructing binomial confidence intervals with near nominal coverage by adding a single imaginary failure or success. *Statistics in Medicine* 2006;25(21):3679-95.
28. Siddiqui O. Statistical methods to analyze adverse events data of randomized clinical trials. *Journal of Biopharmaceutical Statistics* 2009;19(5):889-99. doi: http://dx.doi.org/10.1080/10543400903105463
29. Lancar R, Kramar A, Haie-Meder C. Non-parametric methods for analysing recurrent complications of varying severity. *Statistics in Medicine* 1995;14(24):2701-12.
30. Gong Q, Tong B, Strasak A, et al. Analysis of safety data in clinical trials using a recurrent event approach. *Pharmaceutical Statistics* 2014;13(2):136-44. doi: doi:10.1002/pst.1611
31. Wang J, Quartey G. Nonparametric estimation for cumulative duration of adverse events. *Biometrical Journal* 2012;54(1):61-74.
32. Wang J, Quartey G. A semi-parametric approach to analysis of event duration and prevalence. *Computational Statistics & Data Analysis* 2013;67:248-57. doi: https://doi.org/10.1016/j.csda.2013.05.023
33. Rosenkranz G. Analysis of adverse events in the presence of discontinuations. *Drug Information Journal* 2006;40(1):79-87. doi: 10.1177/009286150604000110
34. Hengelbrock J, Gillhaus J, Kloss S, et al. Safety data from randomized controlled trials: applying models for recurrent events. *Pharmaceutical Statistics* 2016;15(4):315-23. doi: 10.1002/pst.1757
35. Allignol A, Beyersmann J, Schmoor C. Statistical issues in the analysis of adverse events in time-to-event data. *Pharmaceutical Statistics* 2016;15(4):297-305.
36. Nishikawa M, Tango T, Ogawa M. Non-parametric inference of adverse events under informative censoring. *Statistics in Medicine* 2006;25(23):3981-4003.
37. Sogliero-Gilbert G, Ting, N. and Zubkoff, L. . A statistical comparison of drug safety in controlled clinical trials: The Genie score as an objective measure of lab abnormalities. *Therapeutic Innovation & Regulatory Science* 1991;25(1) doi: https://doi.org/10.1177/009286159102500109
38. Berry DA. Monitoring accumulating data in a clinical trial. *Biometrics* 1989;45(4):1197-211.
39. Yao B, Zhu L, Jiang Q, et al. Safety monitoring in clinical trials. *Pharmaceutics* 2013;5(1):94-106.
40. Zhu L, Yao B, Xia HA, et al. Statistical Monitoring of Safety in Clinical Trials. *Statistics in Biopharmaceutical Research* 2016;8(1):88-105. doi: 10.1080/19466315.2015.1117017
41. French JL, Thomas N, Wang C. Using historical data with Bayesian methods in early clinical trial monitoring. *Statistics in Biopharmaceutical Research* 2012;4(4):384-94. doi: 10.1080/19466315.2012.707088

42. Berry SM, Berry DA. Accounting for multiplicities in assessing drug safety: A three-level hierarchical mixture model. *Biometrics* 2004;60(2):418-26. doi: 10.1111/j.0006-341X.2004.00186.x
43. Xia HA, Ma H, Carlin BP. Bayesian hierarchical modeling for detecting safety signals in clinical trials. *Journal of Biopharmaceutical Statistics* 2011;21(5):1006-29.
44. Chen WF, Zhao NQ, Qin GY, et al. A Bayesian Group Sequential Approach to Safety Signal Detection. *Journal of Biopharmaceutical Statistics* 2013;23(1):213-30. doi: 10.1080/10543406.2013.736813
45. McEvoy BW, Nandy RR, Tiwari RC. Bayesian Approach for Clinical Trial Safety Data Using an Ising Prior. *Biometrics* 2013;69(3):661-72.
46. Gould AL. Detecting potential safety issues in clinical trials by Bayesian screening. *Biometrical Journal* 2008;50(5):837-51. doi: 10.1002/bimj.200710469
47. Gould AL. Detecting potential safety issues in large clinical or observational trials by bayesian screening when event counts arise from poisson distributions. *Journal of Biopharmaceutical Statistics* 2013;23(4):829-47. doi: 10.1080/10543406.2013.789887
